# Assessing a novel point-of-care ultrasound training program for rural healthcare providers in Kenya

**DOI:** 10.1186/s12913-018-3196-5

**Published:** 2018-08-06

**Authors:** Grace W. Wanjiku, Gregory Bell, Benjamin Wachira

**Affiliations:** 1 Emergency Medicine, The Warren Alpert Medical School of Brown University, 55 Claverick Street, Suite 100, Providence, RI 02903 USA; 2 Emergency Medicine, Emergency Ultrasound University of Iowa Carver College of Medicine, Iowa City, Iowa USA; 3 Emergency Medicine, Section Head & Interim Clinical Director, Accident & Emergency Department, The Aga Khan University, Nairobi, Kenya

**Keywords:** Point-of-care ultrasound, Training, Rural Kenya

## Abstract

**Background:**

A novel point-of-care ultrasound (PoCUS) training program was developed to train rural healthcare providers in Kenya on the Focused Assessment with Sonography for Trauma (FAST), thoracic ultrasound, basic echocardiography, and focused obstetric ultrasonography. The program includes a multimedia manual, pre-course testing, 1-day hands-on training, post-testing, 3-month post-course evaluation, and scheduled refresher training. This study evaluates the impact of the course on PoCUS knowledge and skills. Competency results were compared based on number of previous training/refresher sessions and time elapsed since prior training.

**Methods:**

Trainees were evaluated using a computer-based, 30 question, multiple-choice test, a standardized observed structured clinical exam (OSCE), and a survey on their ultrasound use over the previous 3 months.

**Results:**

Thirty-three trainees were evaluated at 21 different facilities. All trainees completed the written exam, and 32 completed the OSCE. Nine trainees out of 33 (27.3%) passed the written test. Trainees with two or more prior training sessions had statistically significant increases in their written test scores, while those with only one prior training session maintained their test scores. Time elapsed since last training was not associated with statistically significant differences in mean written test scores. Mean image quality scores (95% confidence interval) were 2.65 (2.37–2.93) for FAST, 2.41 (2.03–2.78) for thoracic, 2.22 (1.89–2.55) for cardiac, and 2.95 (2.67–3.24) for obstetric exams. There was a trend towards increased mean image quality scores with increases in the number of prior training sessions, and a trend towards decreased image quality with increased time elapsed since previous training. Forty percent of trainees reported performing more than 20 scans in the previous 3 months, while 22% reported less than 10 scans in the previous 3 months. Second and third trimester focused obstetric ultrasound was the most frequently performed scan type. Frequency of scanning was positively correlated with written test scores and image quality scores.

**Conclusion:**

This novel training program has the potential to improve PoCUS knowledge and skills amongst rural healthcare providers in Kenya. There is an ongoing need to increase refresher/re-training opportunities and to enhance frequency of scanning in order to improve PoCUS competency.

**Electronic supplementary material:**

The online version of this article (10.1186/s12913-018-3196-5) contains supplementary material, which is available to authorized users.

## Background

Low and moderate-income countries (LMICs) face numerous challenges in the provision of healthcare services to their populations. The World Health Organization (WHO) estimates that 60% of the world’s population does not have access to basic x-ray, computed tomography scanners or other means of tissue imaging in their local health centers [1]. Ultrasonography has demonstrated unique potential for developing health services in LMICs. It is relatively cheap, portable, non-invasive, and easy to operate. Several outcome studies have demonstrated the diagnostic utility of ultrasound in medical, surgical, and obstetric care settings [2, 3].

Recognizing the powerful clinical role of ultrasound, health care leaders have developed point-of-care ultrasound (PoCUS) training programs specifically designed for LMICs [4]. These programs serve healthcare workers in diverse settings including district hospitals [5], rural hospitals [6], and refugee camps [7]. Various models address the issue of limited equipment and instructors by creating local experts who in turn train their peers. This ensures both sustainability and program expansion. Results from these programs have shown that non-traditional sonographers, e.g. generalist physicians, nurses, and mid-level healthcare providers, can demonstrate excellent diagnostic accuracy after short, focused training sessions combined with follow-up evaluation and re-training.

The Kenyan healthcare work force is highly limited, with an estimated 14 doctors per 100,000 patients [8], 60% of whom work in urban areas [9]. Most physicians lack postgraduate training, and radiology specialists are rarely available in rural areas. The bulk of health care in this setting is provided by clinical officers (mid-level providers with a diploma in clinical medicine), nurses, and community workers. Training these rural healthcare providers in basic PoCUS applications has the potential to greatly improve patient care in this setting.

### PoCUS training program in Kenya

A novel PoCUS training program was started in Kenya in 2013 and has since undergone a number of changes to improve quality and effectiveness [10]. The program’s intention was to balance the inherent limitations of rural Kenyan clinical practice with the community’s unique set of healthcare needs. Specifically, the providers do not have ready access to trainers, nor ample time or means to attend extended training. Accordingly, this program places a heavy emphasis on pre-program preparation, and learning is largely self-initiated.

For pre-training, each participant receives an educational manual. The manual originally included a comprehensive amount of information. In December 2013, the manual was revised to include only focused, easily read content with supporting illustrations and diagrams. In April 2014, instructional multimedia videos were embedded into the manual to better demonstrate technique and to provide multiple examples of normal findings and common disease states. The theory behind these changes is that ample knowledge gained prior to a hands-on training would hasten the process of skills acquisition and clinical scanning competency.

Since November 2014, trainees are required to achieve a score of 90% on an online test to be enrolled into the program. After pre-training preparation, trainees then participate in a 1-day supervised hands-on training session. Sessions are conducted in small groups led by certified instructors using human volunteers. Emphasis is placed on maximizing hands-on learning. An observed structured clinical exam (OSCE) is conducted at the end of the initial training session to document baseline skills. Trainees are awarded a PoCUS machine at the end of the first session for use at their facilities to gain competence through practice and patient care.

Follow-up in-facility testing is scheduled 3–4 months after initial training. Trainees that do not pass both the written and OSCE-based skill tests during the facility assessment are invited for refresher training 3–4 months later. The follow-up refresher sessions serve two purposes. First, participants are required to re-study the manual in order to strengthen and improve knowledge retention. Knowledge is assessed using a standardized, 30-question multiple choice question (MCQ) exam. Second, refresher skill training is provided to advance the participants’ expertise through hands-on practice and instruction. Scanning ability is assessed again using a post-course OSCE.

The goal of this study is to assess trainee knowledge and skill after participating in a novel PoCUS training program designed for rural healthcare providers in Kenya and to gather feedback to make improvements to the training program design.

## Methods

### Study design

This is a descriptive analysis of results from an independent, on-site evaluation of healthcare providers who participated in the novel PoCUS training program in Kenya. The study was carried out in January 2015.

### Subjects

Trainees who had previously participated in the novel PoCUS training program were eligible for the study. All eligible trainees were contacted to volunteer for the study. Inclusion criteria were (a) having previously participated in the PoCUS training program and (b) providing informed consent to participate in this study. Exclusion criteria were participants in the PoCUS training program who (a) declined to provide informed consent, (b) had received any form of ultrasound training prior to attending the PoCUS training program (this excluded all radiographers), and/or (c) had not done any scanning since attending the PoCUS training program.

### Setting

The study was conducted at the participants’ current healthcare facilities.

### Data collection

The following baseline statistics were collected from the participants: (a) clinical designation (e.g. clinical officer, nurse, medical officer), (b) date of initial PoCUS training, (c) number and dates of subsequent refresher courses and assessments, and (d) results of all previous assessments. In addition, the participants were asked to fill out a survey (Additional file 1: Appendix S1) on their ultrasound use over the previous 3 months.

### Knowledge and skills testing

To assess their PoCUS knowledge, trainees took a standardized, 30-question multiple choice question (MCQ) exam. Each trainee’s results were compared to his or her score at the time of initial training. A passing score was defined as 27/30 (90%).

A standardized OSCE (Additional file 1: Appendix S2) was used to assess the participants’ clinical skills in the performance of the Focused Assessment with Sonography for Trauma (FAST), which evaluates for free fluid in the pleural, peritoneal, and pericardial cavity. They were also assessed on thoracic ultrasound. The evaluation also included first and second/third trimester scanning skills, i.e. identification of the gestational sac, first trimester dating using crown-rump length, detecting and measuring the fetal heart rate, identifying the presenting part, locating the placenta, and measurement of the head circumference and bi-parietal diameter. This evaluation was performed by an independent investigator who was not previously involved in training of participants, and who was credentialed in PoCUS according to American College of Emergency Physician guidelines.

To verify consistency of OSCE results between examiners, a test of the inter-rater reliability was performed using four course examiners on 11 trainees for each of the applications. The intra-class correlation coefficient was 0.695 (95% confidence interval: 0.375–0.995, *p* < 0.001).

OSCEs were performed on healthy volunteers above the age of 18 years old who were randomly recruited at each testing location. These included a male volunteer, a female volunteer in her first 12 weeks of pregnancy and a female volunteer who was more than 20 weeks pregnant. The trainees recruited volunteers from their own facilities. Pregnant volunteers were recruited ahead of time from routine antenatal clinics, and healthy male volunteers were mostly recruited from hospital staff. The volunteers were fully informed of the study by the investigator prior to signing a consent form. No patients were recruited or involved in the study. No names or other identifying information was collected. The volunteers were informed of the PoCUS findings immediately after the scan. Any volunteer who was found to have any pathology was excluded from participation and immediately referred for formal scanning by a qualified radiologist, and subsequently to the relevant clinician if pathology was confirmed.

### Data analysis

Descriptive and comparative analyses were performed using Excel and SAS 9.4 (SAS Institute Inc. Cary, NC, USA) The paired t-test was used to compare test scores, with an alpha of 0.05 or less considered statistically significant. Spearman’s correlation was used to determine the association between frequency of scanning with test scores and image quality scores.

### Reporting and implementation of results

Results from this study were shared with the convenors of the PoCUS training program and the participants.

### Ethics

This study was approved by the Aga Khan University, Nairobi Research and Ethics Committee (2014/REC-64 (v3)).

## Results

Thirty-three trainees from 21 rural and under-resourced healthcare facilities participated in the written and skills testing. Of these, 15, 9, 7, and 2 trainees had previously completed initial training, 1 refresher session, 2 refresher sessions, and 3 refresher sessions, respectively. All completed the written exam, and 20 completed all items on the OSCE. The remaining 13 trainees were unable to recruit a female volunteer in her first trimester of pregnancy, and therefore they were not tested on two skills: identifying the gestational sac and sagittal view of the uterus in the first trimester.

### PoCUS use in clinical evaluations

Trainees were surveyed on their use of PoCUS in clinical practice. Ninety-four percent (31/33 participants) completed the survey. In the 3 months prior to the study, 22% reported performing fewer than 10 scans, 39% reported performing 10–20 scans, and 39% reported performing > 20 scans (Table 1). Among the High-Use Group (> 20 scans in the prior 3 months) 42% reported performing more than 20 scans in the preceding month.

**Table 1:** Summary of survey results related to frequency of scanning

3 Months Prior (All Participants, *N* = 31)		1 Month Prior (High-Use Group, *N* = 12)	
Frequency	N (%)	Frequency	N (%)
< 10 scans	7(22%)	< 10 scans	1 (8%)
10–20 scans	12 (39%)	10–20 scans	6 (50%)
> 20 scans	12 (39%)	> 20 scans	5 (42%)

The High-Use Group was defined as participants who had performed > 20 scans in the three months prior to the study

Obstetric ultrasounds were the most frequently performed scan type (Table 2).

**Table 2:** Summary of survey results related to types of scans performed in the 1 month prior to the study

Scan Type Performed in Previous Month	
Scan Type	N (%)
Cardiac	2 (7%)
FAST	4(14%)
Pregnancy:	
1st trimester	8 (25%)
2nd/3rd trimester	17(54%)

### Mean written test scores relative to number of prior training sessions

**Fig. 1:**
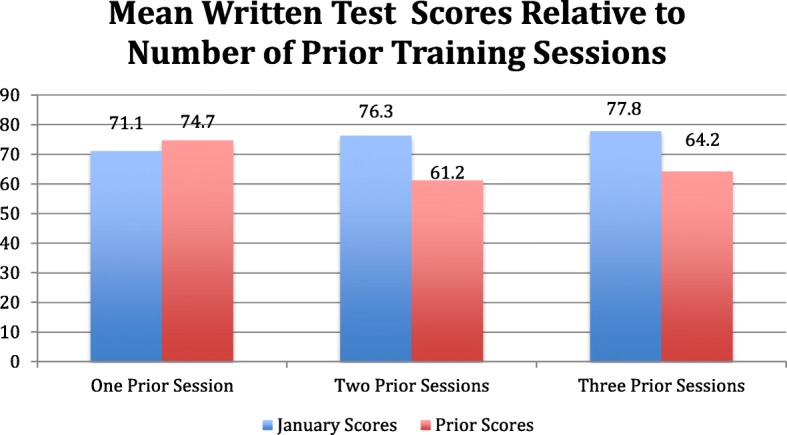
Mean written test scores relative to number of prior training sessions

Twenty-seven percent (9/33) of all trainees passed the written test. Group mean score was 73.6% compared to a prior mean score of 68.2%. For participants with one prior training session (*n* = 15), mean score was 71.1% compared to a prior score of 74.4%, mean difference 3.6% (*p* = 0.33). For participants with two prior training sessions (*n* = 8), mean score was 76.3% compared to 61.2%, which was the mean score from two previous testing sessions. The mean difference was 15.1% (*p* = 0.03). Participants with three or more prior training sessions (*n* = 9) had a mean score of 77.8% compared to 64.2%, which was the combined mean score from three previous testing sessions. The mean difference for this group was 13.5% (*p* < 0.01) (Fig. 1).

### Mean written test scores relative to time elapsed since prior training

**Fig. 2:**
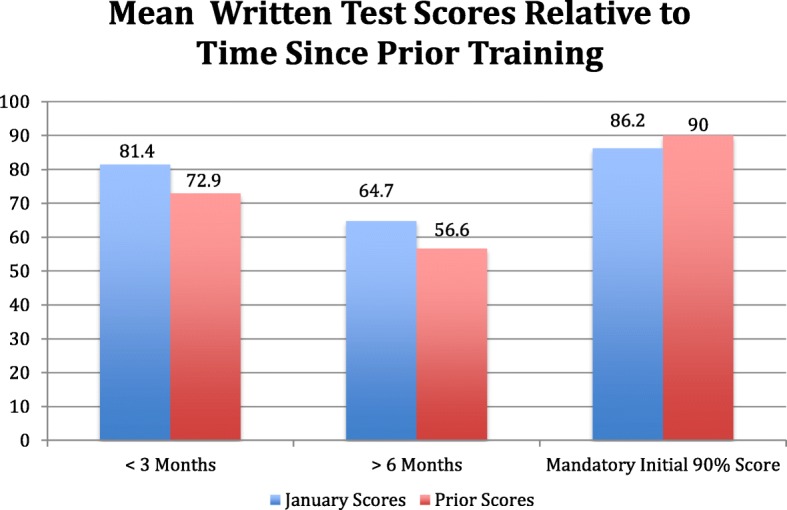
Mean written test scores relative to time elapsed since prior training

The amount of time elapsed since the most recent training was also evaluated for written score differences. The most recent training was considered to be initial training or refresher training. For those trained < 3 months prior (*n* = 7), scores were 81.4% compared to 72.9% previously, mean difference 8.6% (*p* = 0.06). For those trained > 6 months prior to the study (*n* = 17), scores were 64.7% compared to 56.6% previously, mean difference 8.1% (*p* = 0.08). For trainees that were required to obtain a 90% score for initial training (*n* = 8), scores averaged 86.2% with a mean difference of 3.8% (*p* = 0.34) (Fig. 2).

### OSCE image quality scores

Image quality was scored using a standardized scoring system from 0 to 4 [0, no meaningful images; 1, poor, not sufficient for interpretation; 2, good, acceptable for interpretation; 3, excellent, minor suggestions for improvement; 4, outstanding, no suggestions for improvement (Additional file 1: Appendix S2)]. Each trainee was scored on image quality in the performance of FAST, thoracic, basic echocardiography and focused obstetric exams. Thirty-two participants participated in OSCE testing. Mean image quality scores (95% confidence interval) were 2.65 (2.37–2.93) for FAST, 2.41 (2.03–2.78) for thoracic, 2.22 (1.89–2.55) for cardiac, and 2.95 (2.67–3.24) for obstetric ultrasound. Table 3 shows image quality scores for trainees who had one prior, two prior, and three prior training sessions. We observed a trend towards an increase in mean image quality scores with an increase in number of training sessions.

**Table 3:** Image quality scores relative to number of prior training sessions

Prior training sessions	Image Quality Score Mean (95% Confidence Interval)			
	FAST	Thoracic	Cardiac	Obstetrics
1	2.60 (2.24–2.96)	2.10 (1.73–2.61)	2.07 (1.65–2.48)	2.73 (2.31–3.15)
2	2.37 (1.52–3.23)	2.31(1.09–3.54)	2.19 (1.29–3.08)	2.95 (2.21–3.69)
3	2.98 (2.43–3.52)	2.89 (2.18–3.60)	2.50 (1.68–3.32)	3.33 (2.84–3.83)

The effect of time elapsed since the most recent prior training on image quality scores was evaluated. Similar to the written score evaluation, the most recent training was considered to be either initial training or refresher training. Table 4 shows image quality scores relative to time elapsed since prior training. We observed a trend towards a decrease in image quality scores with an increase in time elapsed since prior training.

**Table 4:** Image quality scores relative to time elapsed since prior training

Months since prior training	Image Quality Score Mean (95% Confidence Interval)			
	FAST	Thoracic	Cardiac	Obstetrics
< 3	3.17 (2.61–3.73)	3.07 (2.34–3.80)	2.64 (1.65–3.63)	3.64 (3.37–3.91)
> 6	2.29 (1.87–2.72)	2.15 (1.55–2.75)	1.91 (1.44–2.39)	2.74 (2.34–3.13)

Frequency of scanning was positively correlated with written test scores (Spearman correlation coefficient 0.19, *p* = 0.30) and image quality scores (Spearman correlation coefficient 0.26, *p* = 0.16).

### Challenges faced by trainees

A questionnaire completed by all the trainees at the end of each training session revealed several prevalent program challenges. One of the most common was the request for more supervised training beyond the scheduled training sessions, especially for obstetric applications. Some trainees requested on-site instruction, though this is not logistically feasible for trainers to travel to multiple facilities. While most trainees felt that the applications taught were appropriate for their patient populations, some requested training on the use of ultrasound for procedural guidance. One final consistent request was that printers be provided so that clinicians could provide patients with images to increase patient confidence and satisfaction with care.

## Discussion

This study highlights several strengths of the novel PoCUS training program as well as limitations and opportunities for further work. We found a wide variation in the frequency of ultrasound use by the trainees. Despite being awarded ultrasound machines upon successful completion of their first training session, the overall frequency of use remains low.

The most frequently used application (79%) was second/third trimester obstetrics. This finding corroborates other findings from the region that have shown that the primary use of PoCUS in sub-Saharan Africa is in the management of pregnant women [2, 3]. Trainees achieved the highest image quality scores in the obstetrics category, followed by FAST exams. This finding is likely secondary to frequency of ultrasound performance, given that obstetric ultrasound was most frequently performed, followed by the FAST exam. We found a weakly positive correlation between frequency of scanning and test scores, and a stronger positive correlation between frequency of scanning and image quality scores. We expect that increase in frequency of ultrasound use will contribute towards improved image quality.

The number of training sessions was evaluated as a factor affecting performance. Trainees with two or three prior training sessions were found to have statistically significant increases in their MCQ test scores. We also noted a trend towards increased image quality scores with increase in number of prior training sessions.

In addition, we considered the time elapsed since the previous training session. Trainees who had received training more recently (< 3 months) performed better than those who received training > 6 months prior. We also noted a trend towards decreased image quality with increase in time since the previous training. This highlights the need to increase opportunities for re-training, especially for clinicians who are unable to return to the capital city for refresher training. Arranging training sessions either in-facility or in nearby towns will be useful.

Limitations of this study include the fact that frequency of ultrasound use was based on a combination of ultrasound logs and self-report. Some clinicians keep an up-to-date log while others do not, and this limited our data. Our data is also limited by a small sample size. Future work will focus on increasing opportunities for hands-on training and feedback. We will also explore interventions aimed at enhancing frequency of scanning, which we believe will increase the quality of ultrasound performance by our trainees.

## Conclusion

Achieving an effective educational PoCUS program for clinicians in rural, resource-limited settings is a logistically difficult endeavor. Each aspect including course design, development of training resources, availability of expert trainers, knowledge and skill evaluation requires careful implementation and ongoing assessment [11]. Our program deals with these challenges by developing a locally relevant curriculum and focusing on self-directed learning using multi-media educational material that is available offline. Since November 2014, trainees are required to pass a preliminary exam with a near-perfect mark prior to participating in hands-on sessions. This encourages not only a thorough knowledge of the material but also critical levels of interpretation and problem solving in order to get the most out of practical training. The focus of the initial and refresher training sessions is high-intensity, hands-on learning and rigorous follow-up assessment of competency.

As reported in this paper, we are performing on-site evaluations to offer practical advice to trainees about applying ultrasound to their practice, to assess their ongoing knowledge and proficiency in practical skills, to elicit feedback about the program, and to offer further hands-on training tips. Going forward, we will work to increase opportunities for hands-on skill training and modeling and create more opportunities for quality assessment and feedback. Additionally, we will design and evaluate interventions geared to increasing the frequency and quality of PoCUS performance. Our overall goal is to advocate for the continued development of PoCUS as a tool to assist rural, under-resourced healthcare providers in their clinical decision-making.

## Additional file

Additional file 1: **Appendix S1.** Ultrasound use assessment. Survey of ultrasound use within the last 3 months. **Appendix S2.** Observed Structured Clinical Exam Assessment form. OSCE evaluation criteria. (DOCX 44 kb)

## Abbreviations

CO: Clinical Officer
FAST: Focused Assessment with Sonography for Trauma
HIV: Human Immuno-deficiency Virus
LMICs: Low and Middle Income Countries
MCQ: Multiple Choice Question
OSCE: Observed Structured Clinical Exam
PoCUS: Point-of-Care Ultrasound
WHO: World Health Organization
