# Supplementary material for: Assessing a novel point-of-care ultrasound training program for rural healthcare providers in Kenya
Source: BMC Health Serv Res. 2018 Aug 6;18:607. doi: 10.1186/s12913-018-3196-5 (PMC6091199; doi:10.1186/s12913-018-3196-5)
Supplement: Supplementary file 1 — Appendix S1. Ultrasound use assessment. Survey of ultrasound use within the last 3 months. Appendix S2. Observed Structured Clinical Exam Assessment form. OSCE evaluation criteria. (DOCX 44 kb) [file 12913_2018_3196_MOESM1_ESM.docx]

**Appendix S1**

# Ultrasound Use Assessment

Dear Clinician,

This survey is meant to understand your use of ultrasound over the past three months.

Your responses to this survey will help us to better understand your needs and how we may change our training to better meet your needs. Your completion of this survey is completely voluntary and you are under no obligation to complete it. You will continue to receive the training and support regardless of whether you complete this survey or not.

The data collected will be used as part of a research publication and your completion of this survey will constitute your consent for this purpose. Please contact us at [benjawambugu@yahoo.com](mailto:gregrussellbell@gmail.com) with any questions in regards to this project. Thank you in advance for your participation.

1. Did you receive any previous ultrasound use training prior to attending the PoCUS training program?

□ Yes □ No

1. How many ultrasounds have you performed yourself over the past 3 months?

□ 0 □ 1-5 □ 6-9 □ 10-20 □ >20

1. How many ultrasounds have you performed yourself over the past month?

□ 0 □ 1-5 □ 6-9 □ 10-20 □ >20

1. How many ultrasounds have you performed yourself over the past week?

□ 0 □ 1-5 □ 6-9 □ 10-20 □ >20

1. How many of each ultrasound type do you estimate to have performed over the past month?

| EFAST | □ 0 | □ 1-5 | □ 6-9 | □ 10-20 | □ >20 |
| --- | --- | --- | --- | --- | --- |
| Echocardiography | □ 0 | □ 1-5 | □ 6-9 | □ 10-20 | □ >20 |
| OB – 1^st^ trimester | □ 0 | □ 1-5 | □ 6-9 | □ 10-20 | □ >20 |
| OB -2^nd^ and 3^rd^ trimester | □ 0 | □ 1-5 | □ 6-9 | □ 10-20 | □ >20 |
| Other: | □ 0 | □ 1-5 | □ 6-9 | □ 10-20 | □ >20 |
|  | □ 0 | □ 1-5 | □ 6-9 | □ 10-20 | □ >20 |
|  | □ 0 | □ 1-5 | □ 6-9 | □ 10-20 | □ >20 |
|  | □ 0 | □ 1-5 | □ 6-9 | □ 10-20 | □ >20 |
|  | □ 0 | □ 1-5 | □ 6-9 | □ 10-20 | □ >20 |
|  | □ 0 | □ 1-5 | □ 6-9 | □ 10-20 | □ >20 |

1. How many of each ultrasound type do you estimate to have performed over past week?

| EFAST | □ 0 | □ 1-5 | □ 6-9 | □ 10-20 | □ >20 |
| --- | --- | --- | --- | --- | --- |
| Echocardiography | □ 0 | □ 1-5 | □ 6-9 | □ 10-20 | □ >20 |
| OB – 1^st^ trimester | □ 0 | □ 1-5 | □ 6-9 | □ 10-20 | □ >20 |
| OB -2^nd^ and 3^rd^ trimester | □ 0 | □ 1-5 | □ 6-9 | □ 10-20 | □ >20 |
| Other: | □ 0 | □ 1-5 | □ 6-9 | □ 10-20 | □ >20 |
|  | □ 0 | □ 1-5 | □ 6-9 | □ 10-20 | □ >20 |
|  | □ 0 | □ 1-5 | □ 6-9 | □ 10-20 | □ >20 |
|  | □ 0 | □ 1-5 | □ 6-9 | □ 10-20 | □ >20 |
|  | □ 0 | □ 1-5 | □ 6-9 | □ 10-20 | □ >20 |
|  | □ 0 | □ 1-5 | □ 6-9 | □ 10-20 | □ >20 |

1. How many days during the past month have you had any of the following problems with your ultrasound machine?

| Did not function properly? | □ 0 days | □ 1-7 days | □ 8-15 days | □ >15 days |
| --- | --- | --- | --- | --- |
| Ran out of supplies? | □ 0 days | □ 1-7 days | □ 8-15 days | □ >15 days |
| Problems with image quality? | □ 0 days | □ 1-7 days | □ 8-15 days | □ >15 days |
| Screen was not working? | □ 0 days | □ 1-7 days | □ 8-15 days | □ >15 days |
| Problem with a transducer? | □ 0 days | □ 1-7 days | □ 8-15 days | □ >15 days |

1. In the instance when ultrasound machine did not function, were you able to fix problem:

| □ On the same day |
| --- |
| □ Within the week |
| □ Within the month |
| □ It’s still broken |

1. In the past month, has the use of ultrasound changed your clinical patient management?

| □ Never |
| --- |
| □ yes, less than half of the time |
| □ yes, about half of the time |
| □ yes, more than half of the time |

1. In the past month, how frequently has the use of ultrasound led you to refer a patient to a referral hospital?

| □ Never |
| --- |
| □ yes, less than half of the time |
| □ yes, about half of the time |
| □ yes, more than half of the time |

1. In the past month, how frequently has the use of ultrasound led you to perform a procedure on a patient?

| □ Never |
| --- |
| □ yes, less than half of the time |
| □ yes, about half of the time |
| □ yes, more than half of the time |

1. Of the following, please list the top three applications that have had an impact on patient management over the last month:

1. ____________________ 2.____________________ 3.____________________

□ EFAST □ Echocardiography □ OB -1^st^ trimester □ OB - 2^nd^ & 3^rd^ trimester

□ Other: ______________________________________________________________

1. On a scale from 1-5, please rate your level of comfort in acquiring images with the ultrasound machine (1= not comfortable at all; 5= very comfortable):

□ 1 □ 2 □ 3 □ 4 □ 5

1. On a scale from 1-5, please rate your level of comfort in interpreting each of the following ultrasound exams (1= not comfortable at all; 5=very comfortable):

| EFAST | □ 1 | □ 2 | □ 3 | □ 4 | □ 5 |
| --- | --- | --- | --- | --- | --- |
| Echocardiography | □ 1 | □ 2 | □ 3 | □ 4 | □ 5 |
| OB – 1^st^ trimester | □ 1 | □ 2 | □ 3 | □ 4 | □ 5 |
| OB – 2^nd^ and 3^rd^ trimester | □ 1 | □ 2 | □ 3 | □ 4 | □ 5 |
| Other | □ 1 | □ 2 | □ 3 | □ 4 | □ 5 |
|  | □ 1 | □ 2 | □ 3 | □ 4 | □ 5 |
|  | □ 1 | □ 2 | □ 3 | □ 4 | □ 5 |
|  | □ 1 | □ 2 | □ 3 | □ 4 | □ 5 |

1. How have your patients responded to your use of ultrasound?

□ they have responded positively

□ they have been worried about its use

□ I am not sure

1. Do you agree with the following statement?

The use of ultrasound is useful to my practice:

| □ Yes, strongly agree | □ Yes, somewhat agree | □ No opinion |
| --- | --- | --- |
| □ No, somewhat disagree | □ No, strongly disagree |  |

1. Please feel free to include questions or comments on any questions in the space below:

**Appendix S2**

Dear Clinician,

Welcome to the Observed Structured Clinical Exam. The purpose of this exercise is to assess your abilities to use the ultrasound machine to acquire images and your ability to interpret the images that you acquire. All of the images that we will test you on are standard applications of point-of-care ultrasound. The Evaluator will observe you in many areas of your use and rate your skills on the following items:

1. General use of ultrasound
2. Communication with patient
3. Technique in use of ultrasound
4. Quality of images and image interpretation

The data collected will be used as part of a research publication and your participation in this OSCE will constitute your consent for this purpose. Thank you in advance for your participation.

**Instructions for Evaluator**

**Pre-scan**

1. The Evaluator reads the following:

“In this OSCE we will be assessing your performance of all aspects of point-of-care ultrasound. Prior to the scan we will assess how you approach a patient, how you set up the machine, and your attention to hygiene and infection control. During the scanning we will assess your ability to acquire and interpret images based on the criteria that you have learned throughout the course and your interaction with the patient. After the scan we will assess how well you communicate with the patient and attend to them.”

1. The Evaluator and Trainee approach a patient and ask permission to scan them. Patients must give verbal consent prior to initiation of the OSCE. The Trainee should be blinded to clinical information as best as possible.
2. The Trainee sets up the machine. The Evaluator assesses for all of the aspects on the Pre-scan score sheet. If any are not done correctly by the Trainee, they are attended to BEFORE scanning occurs (i.e. enter the patient information correctly, clean probes, etc.)

**Scan**

1. For each required view, the Evaluator reads the name of the view and the Trainee:
   1. Places the probe on the patient (Evaluator scores PO, OR)
   2. Adjusts the depth and gain and other machine settings to optimize the image
   3. Demonstrates knowledge of anatomy by pointing out relevant structures and relationships. The Evaluator can ask questions here to assure complete comprehension (Evaluator scores AN)
   4. Interprets image in line with point-of-care ultrasound questions. (Evaluator scores IN)
   5. Freezes an image with representative of findings. (Evaluator scores IM)

**Post-Scan**

1. After scanning is complete, the Trainee cleans up, and tells the patient what was seen (Evaluator scores CO). They thank the patient and leave. The Evaluator scores the remainder of the post-scan questions.
2. The Evaluator gives direct feedback to the Trainee, reviewing all components of the score sheet and offering suggestions for improvement.

**Observed Structured Clinical Exam Assessment Form**

**Trainee Name: ____________________________________________________________**

**Designation: ______________________________________________________________**

**Date: ____________________________________________________________________**

| Pre-scan |  | | | | | Score |
| --- | --- | --- | --- | --- | --- | --- |
| 1 | Turned on machine (Y/N) | | | | |  |
| 2 | Positioned machine properly (Y/N) | | | | |  |
| 3 | Positioned patient properly (Y/N) | | | | |  |
| 5 | Cleaned hands prior to scan (Y/N) | | | | |  |
| 6 | Cleaned probes prior to scan (Y/N) | | | | |  |
| Scanning | | PO | OR | AN | IN | IM |
| Cardiac-Subxiphoid | |  |  |  |  |  |
| Cardiac-PSL | |  |  |  |  |  |
| FAST-RUQ | |  |  |  |  |  |
| FAST-LUQ | |  |  |  |  |  |
| FAST-Suprapubic | |  |  |  |  |  |
| Thoracic-Pneumothorax | |  |  |  |  |  |
| Thoracic-Pleural Fluid | |  |  |  |  |  |
| Ob – Gestational Sac | |  |  |  |  |  |
| Ob – Foetal Heart Rate | |  |  |  |  |  |
| Ob - Sagittal Uterus | |  |  |  |  |  |
| Ob - Presentation | |  |  |  |  |  |
| Ob – Placental Location | |  |  |  |  |  |
| Ob – Head Circumference Measurement | |  |  |  |  |  |
| Ob – Bi-parietal Diameter Measurement | |  |  |  |  |  |
| Post-scan |  | | | | | Score |
| 1 | Assured patient was comfortable during scan (Y/N) | | | | |  |
| 2 | Documented findings appropriately (DO) | | | | |  |
| 3 | Communicated well with patient (CO) | | | | |  |
|  | **Written Score** | | | | |  |

**Scoring System**

Pre-scan and most of Post-scan, unless otherwise specified

1=Yes, done correctly

0=No, not done correctly

PO: POsition

1=the probe was placed on the correct position on the patient’s body

0=the probe was placed on the incorrect position

OR: ORientation

1=the selection marker was pointing in the correct direction

0=the selection marker was pointing in the incorrect direction

AN: ANatomy

3=correctly identified and named ALL structures pertinent to image

2=correctly identified and named SOME structures pertinent to image, did not know all

1=correctly identified and named some structures but INCORRECTLY identified or named others

0=could not identify or name pertinent structures or named them all incorrectly

IN: INterpretation

3=correctly acquired and interpreted images to answer ALL relevant point-of-care questions

2=correctly acquired images and was able to interpret SOME but not all relevant point-of-care questions

1=correctly acquired images but INCORRECTLY interpreted some of them

0=did not acquire images sufficient for interpretation to answer point-of-care question

IM: IMage quality

4=outstanding images, no suggestions for improvement

3=excellent images, minor suggestions for improvement

2=good images, acceptable for interpretation

1=poor images, some anatomy discernible but not sufficient for interpretation

0=no meaningful image generated

CO=COmmunication

3=Excellent communication, communicated well with the patient/family, explaining and not overstating findings

2=Good communication, communicated some findings with the patient/family

1=Poor communication, explained some findings incorrectly or overstated findings

0=No communication
